# High resolution magic angle spinning 1H NMR of childhood brain and nervous system tumours

**DOI:** 10.1186/1476-4598-8-6

**Published:** 2009-02-10

**Authors:** Martin Wilson, Nigel P Davies, Marie-Anne Brundler, Carmel McConville, Richard G Grundy, Andrew C Peet

**Affiliations:** 1 Cancer Sciences, University of Birmingham, Birmingham, UK; 2 Birmingham Childrens Hospital NHS Foundation Trust, Birmingham, UK; 3 Medical Physics and Imaging, University Hospital Birmingham Foundation Trust, Birmingham, UK; 4 Childrens Brain Tumour Research Centre, Nottingham University Hospitals, Nottingham, UK

## Abstract

**Background:**

Brain and nervous system tumours are the most common solid cancers in children. Molecular characterisation of these tumours is important for providing novel biomarkers of disease and identifying molecular pathways which may provide putative targets for new therapies. 1H magic angle spinning NMR spectroscopy (1H HR-MAS) is a powerful tool for determining metabolite profiles from small pieces of intact tissue and could potentially provide important molecular information.

**Methods:**

Forty tissue samples from 29 children with glial and primitive neuro-ectodermal tumours were analysed using HR-MAS (600 MHz Varian gHX nanoprobe). Tumour spectra were fitted to a library of individual metabolite spectra to provide metabolite values. These values were then used in a two tailed t-test and multi-variate analysis employing a principal component analysis and a linear discriminant analysis. Classification accuracy was estimated using a leave-one-out analysis and B632+ bootstrapping.

**Results:**

Glial tumours had significantly (two tailed t-test p < 0.05) higher creatine and glutamine and lower taurine, phosphoethanolamine, phosphorylcholine and choline compared with primitive neuro-ectodermal tumours. Classification accuracy was 90%. Medulloblastomas (n = 9) had significantly (two tailed t-test p < 0.05) higher creatine, glutamine, phosphorylcholine, glycine and scyllo-inositol than neuroblastomas (n = 7), classification accuracy was 94%. Supratentorial primitive neuro-ectodermal tumours had metabolite profiles in keeping with other primitive neuro-ectodermal tumours whilst ependymomas (n = 2) had metabolite profiles intermediate between pilocytic astrocytomas (n = 10) and primitive neuro-ectodermal tumours.

**Conclusion:**

HR-MAS identified key differences in the metabolite profiles of childhood brain and nervous system improving the molecular characterisation of these tumours. Further investigation of the underlying molecular pathways is required to assess their potential as targets for new agents.

## Background

Childhood brain and nervous system tumours are the most common solid cancers of childhood. They comprise a diverse set of diseases from the highly malignant to the histologically 'benign' with a corresponding variety of treatments, prognoses and outcomes. Improvements in outcome have not matched those in other forms of childhood cancer and new methods are required to understand the biology of these tumours and develop novel approaches to therapy.

Currently the treatment of these tumours is largely determined through categorization of the cases by histopathology, location, stage and patient age. The most common high grade tumours can be categorised as primitive neuroectodermal tumours (PNETs) based on their histopathological appearance [1]. PNETs are embryonal tumours and have subgroups which occur in various locations of the brain, the sympathetic nervous system and the eye. Neuroblastoma, arises from the sympathetic nervous system and often presents with metastases at diagnosis and is particularly challenging to treat. Intracranial PNETs are all WHO grade IV tumours which have metastatic potential and follow an aggressive clinical course.

Medulloblastomas occur in the cerebellum, pineoblastomas in the pineal gland and supratentorial PNETs in other supratentorial regions. They are all poorly differentiated tumours with closely related histopathology. Despite their many similarities, treatment is tailored to the specific sub-type of tumour and improved characterization is an important objective.

Other childhood brain tumours are diverse in terms of histopathology, grade and clinical behaviour. In addition to PNETs, brain tumours can belong to another common histopathological category known as glial tumours. These tumours are thought to arise from the supportive tissue of the brain, glia. Astrocytomas, many of which are WHO grade I, are the most common example of these tumours in the brain. Ependymomas are locally aggressive tumours which are predominantly WHO grade 2 and 3.

Although histopathology is an important method of characterizing tumours and is the main method currently for providing a diagnosis, it is not always straightforward to distinguish between different tumour types using this method and the development of new techniques may improve characterisation and diagnosis in difficult cases. Furthermore histopathology is often a poor predictor of tumour behaviour and response to treatment. The improved characterization of these tumour types through the discovery of novel biomarkers is an important step in optimizing treatment for individual patients.

Tumour genetics is emerging as an important adjunct to histopathological diagnosis and clinical indicators in determining prognosis and stratifying treatment. Amplification of the MYCN oncogene is already used clinically as a prognostic marker to stratify treatment in neuroblastoma and cMyc has been linked to a more aggressive phenotype in medulloblastoma [2,3]. Furthermore, gene expression profiling has been highly successful in subcategorizing the different subtypes of PNETs and has led to the discovery of prognostic markers [4]. Through this process, specific molecular pathways are being identified for specific tumours leading to the discovery of potential targets for new therapeutic agents.

With the identification of specific patterns of gene expression, there is increasing interest in probing the downstream molecular pathways related to these changes. One strategy which has emerged as being of particular interest is the broad sampling of metabolite levels as a measure of tumour metabolism, a strategy commonly termed metabolomics. 1H nuclear magnetic resonance (NMR) spectroscopy can measure the concentration of a range of metabolites and is a particularly powerful tool for measuring metabolite profiles. Several studies have used NMR to measure metabolite profiles from chemical extracts of excised brain tumour tissue and found that specific metabolites differ between brain tumour and healthy brain [5], low grade and high grade astrocytic tumours [6,7], and glioblastoma and metastatic tumours. It has been shown more recently that total choline correlates with tumour progression [8]. The majority of extract studies on brain tumour tissue have focused on adult astrocytomas however a study of pediatric posterior fossa tumours [9] showed that medulloblastomas could be distinguished from astrocytomas by their metabolite profile.

A variant of the NMR technique known as high resolution magic angle spinning NMR (HR-MAS) allows metabolite profiling of intact tissue. The technique provides high resolution data on small (5–30 mg) inhomogeneous samples making it ideal for the study of tissue [10]. The technique has had success in characterising a range of tissues including diseased brain [11], breast tumour [12,13], cervical [14], liver tumour [15,16], primary [17-19] and metastatic [20] adult brain tumours and paediatric brain tumours [21,22]. HR-MAS results also show a good correlation with in vivo metabolite profiles measured by magnetic resonance spectroscopy in patients [21,23].

Recently, semi-automated methods have been developed for accurately quantitating metabolite concentrations from 1H HR-MAS spectra [24,25]. Multivariate techniques such as principle component analysis (PCA) and linear discriminant analysis (LDA) can be used to analyse these metabolite concentrations with the goal of improving tumour characterisation and classification [26,27]. The combination of minimal sample preparation, speed of collecting HR-MAS data and automated analysis/classification, gives the potential for this strategy to provide a rapid diagnostic aid. Current methods used for rapid diagnosis such as frozen section analysis have a low accuracy and HR-MAS provides a potential method to improve this. However, more importantly, the analysis of metabolite profiles gives the opportunity to identify key molecular pathways to improve our understanding of tumour biology and provide new targets for novel therapeutic agents.

In this study we use 1H HR-MAS of intact tissue to explore childhood PNETs and glial tumours. The initial aim is to ascertain whether these tumours form distinct groups according to their metabolite profiles and, if so, to determine the characteristic metabolite profiles of the tumour groups. For PNETs and glial tumours there are sufficient numbers of cases in this study to estimate the accuracy of classification by this method. Numbers in specific tumour groups are smaller but a preliminary analysis has been performed on the three main tumour types grade 1 astrocytomas, medulloblastomas and neuroblastomas. For the other tumour groups, the relationship of these cases to the larger groups has been explored.

## Results

### Comparison of intra and inter tumour variability

An analysis of the neuroblastoma tumours showed that 10 of the 17 metabolites measured have a significantly (z-test) smaller variability between tumour samples from the same patient than between tumour samples taken from different patients. No metabolites showed a significantly greater intra-tumour than inter-tumour variability. All subsequent analyses were performed with a dataset containing the mean metabolite values for each tumour to avoid bias due to multiple samples being available for some tumours.

### Comparison Between PNET and Glial Tumours

**Figure 1:**
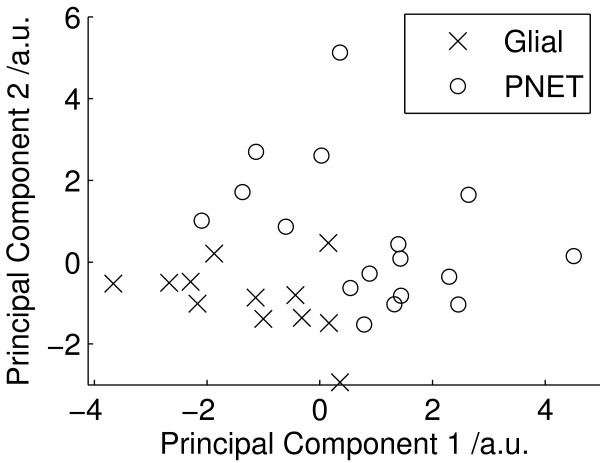
**PCA for all tumours**. PCA scores of the fitted metabolite quantities for all tumours.

**Figure 2:**
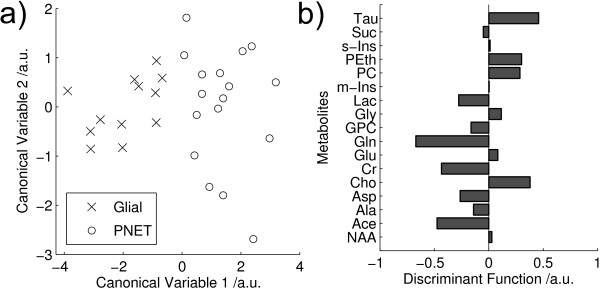
**LDA PNET vs glial**. Linear discriminant analysis of PNET vs glial tumours performed on the first 3 principal components of fitted metabolite quantities showing a) Discriminant Function (DF) scores and b) coefficients of the first DF. Tau – Taurine, Suc – Succinate, s-Ins – scyllo inositol, PEth – phosphorylethanolamine, PC – phosphorylcholine, m-Ins – myo-inositol, Lac – lactate, Gly – glycine, GPC – glycerophosphorylcholine, Gln – glutamine, Glu – glutamate, Cr – creatine, Cho – choline, Asp – aspartate, Ala – alanine, Ace – aspartate, NAA – N-acetylaspartate. (classifier accuracy: 90%).

Mean metabolite values for all PNET and glial tumours are shown in Table 1. Statistically significant differences (2 tailed t-test, p < 0.05) between PNET and glial samples were found in 6 of the 17 metabolites. Higher taurine, phosphoethanolamine, phosphorylcholine and choline together with lower creatine and glutamine were found to be the important discriminators of PNETs from glial tumours. A principal component analysis performed on all fitted metabolite quantities is shown in Figure 1. The plot shows a good splitting between PNET and glial tumours using the first two principal components. The first and second principal components accounted for 20% and 15% of the variance respectively. Linear discriminant analysis was performed on the first 3 principle component (PC) scores and provides complete separation of the two groups in the first discriminant function, Figure 2a. The corresponding metabolite coefficients are shown in Figure 2b and are dominated by the metabolites found to be significantly different on the t-tests. The classification accuracy was 90% with an error rate of 18%.

**Table 1:** Univariate statistics Glial vs PNET.

Metabolite	Glial		PNET		Two-tailed t-test
	Mean	SE	Mean	SE	P value
NAA	0.80	0.20	0.90	0.27	-
Ace	0.17	0.04	0.12	0.02	-
Ala	1.54	0.23	1.42	0.17	-
Asp	0.55	0.14	0.28	0.07	-
Cho	0.51	0.05	0.74	0.08	< 0.05
Cr	2.58	0.45	1.52	0.22	< 0.05
Glu	2.40	0.34	3.29	0.43	-
Gln	5.10	0.50	2.04	0.38	< 0.0001
GPC	0.79	0.11	0.60	0.17	-
Gly	1.44	0.20	2.08	0.35	-
Lac	12.8	1.10	10.1	0.90	-
m-Ins	3.32	0.79	2.20	0.34	-
PC	0.67	0.05	1.75	0.37	< 0.05
PEth	0.17	0.41	2.25	0.30	< 0.01
s-Ins	0.08	0.19	0.33	0.05	-
Suc	0.08	0.02	0.08	0.02	-
Tau	0.95	0.21	2.38	0.35	< 0.01

Mean and standard error (SE) of metabolite levels for glial and PNET tissue with statistical significance levels from two-tailed students t-tests assuming unequal variance shown for significant metabolites. All metabolite quantities were divided by the fitted spectral area between 0.5 and 4.5 ppm. Tau – Taurine, Suc – Succinate, s-Ins – scyllo inositol, PEth – phosphorylethanolamine, PC – phosphorylcholine, m-Ins – myo-inositol, Lac – lactate, Gly – glycine, GPC – glycerophosphorylcholine, Gln – glutamine, Glu – glutamate, Cr – creatine, Cho – choline, Asp – aspartate, Ala – alanine, Ace – aspartate, NAA – N-acetylaspartate.

### Comparison Between Medulloblastoma and Neuroblastoma Tumours

**Figure 3:**
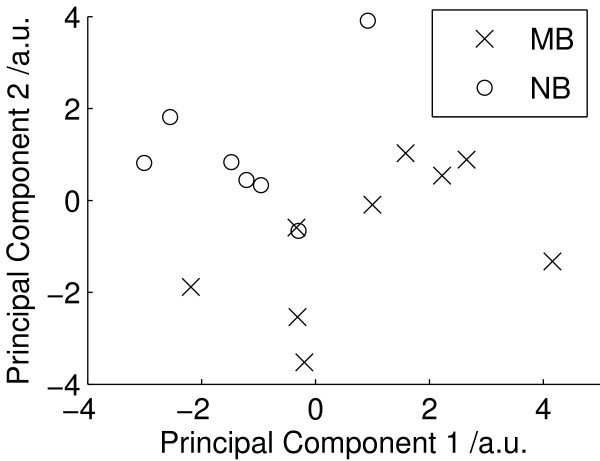
**PCA NB vs MB**. PCA scores plot for neuroblastoma and medulloblastoma cases.

**Figure 4:**
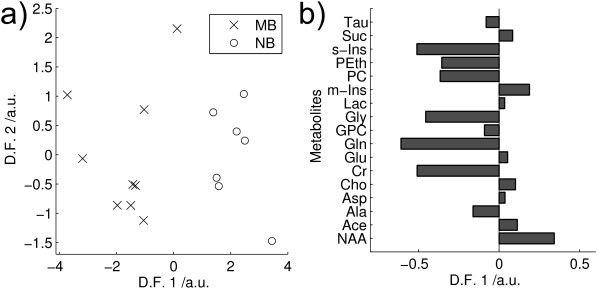
**LDA MB vs NB**. Linear discriminant analysis of Medulloblastoma vs Neuroblastoma performed on the first 5 principal components of the fitted metabolite quantities, showing a) Discriminant function (DF) scores and b) DF 1 metabolite coefficients. Tau – Taurine, Suc – Succinate, s-Ins – scyllo inositol, PEth – phosphorylethanolamine, PC – phosphorylcholine, m-Ins – myo-inositol, Lac – lactate, Gly – glycine, GPC – glycerophosphorylcholine, Gln – glutamine, Glu – glutamate, Cr – creatine, Cho – choline, Asp – aspartate, Ala – alanine, Ace – aspartate, NAA – N-acetylaspartate. (classifier accuracy: 94%).

Mean metabolite quantities for medulloblastoma and neuroblastoma are shown in Table 2. Significant differences (2 tailed t-test, p < 0.05) were found in 5 of the 17 metabolites with medulloblastomas having higher creatine, glutamine, phosphorylcholine, glycine and scyllo-inositol. The PCA scores plot (Figure 3) shows a near complete separation between the medulloblastomas and neuroblatomas. LDA was performed on the first 5 principal components and the scores plot is given in Figure 4a. The first discriminant function gives a complete separation of the groups and the metabolite coefficients of this function are given in Figure 4b. Apart from the 5 metabolites which are significantly different between the tumours, high phosphoethanolamine is also found to be an important discriminant of medulloblastoma. Taurine is neither significantly different between the tumour groups nor an important discriminator between the tumour groups. The classifier accuracy was 94% with an error rate of 19%.

**Table 2:** Univariate statistics Astro vs MB vs NB. Mean and standard error (SE) of metabolite levels for astrocytoma G1 (Astro) (N = 10), medulloblastoma (MB) (N = 9) and neuroblastoma (NB) (N = 7) tissue.

Metabolite	Astro		MB		NB		Two-tailed t-test (MB vs NB)
	Mean	SE	Mean	SE	Mean	SE	P value
NAA	0.90	0.29	0.66	0.22	1.26	0.48	-
Ace	0.19	0.06	0.09	0.03	0.17	0.06	-
Ala	1.63	0.51	1.48	0.49	1.25	0.47	-
Asp	0.54	0.17	0.22	0.07	0.32	0.12	-
Cho	0.52	0.17	0.68	0.23	0.81	0.30	-
Cr	2.45	0.77	2.05	0.68	0.96	0.36	< 0.01
Glu	2.57	0.81	2.90	0.97	3.50	1.32	-
Gln	5.36	1.69	3.15	1.05	0.61	0.23	< 0.001
GPC	0.81	0.25	0.60	0.20	0.61	0.23	-
Gly	1.57	0.50	2.83	0.94	1.29	0.49	< 0.05
Lac	13.5	4.27	9.29	3.10	11.2	4.24	-
m-Ins	2.25	0.71	1.72	0.57	2.72	1.03	-
PC	0.66	0.21	2.45	0.82	0.87	0.33	< 0.05
PEth	1.19	0.38	2.67	0.89	1.61	0.61	-
s-Ins	0.35	0.11	0.42	0.14	0.23	0.09	< 0.05
Suc	0.09	0.03	0.07	0.02	0.09	0.03	-
Tau	0.90	0.28	2.61	0.87	1.92	0.73	-

Statistically significant differences between medulloblastomas and neuroblastomas resulting from two-tailed students t-tests assuming unequal variances are indicated. All metabolite quantities were divided by the fitted spectral area between 0.5 and 4.5 ppm. Tau – Taurine, Suc – Succinate, s-Ins – scyllo inositol, PEth – phosphorylethanolamine, PC – phosphorylcholine, m-Ins – myo-inositol, Lac – lactate, Gly – glycine, GPC – glycerophosphorylcholine, Gln – glutamine, Glu – glutamate, Cr – creatine, Cho – choline, Asp – aspartate, Ala – alanine, Ace – aspartate, NAA – N-acetylaspartate.

### Comparison Between All Tumour Types

**Figure 5:**
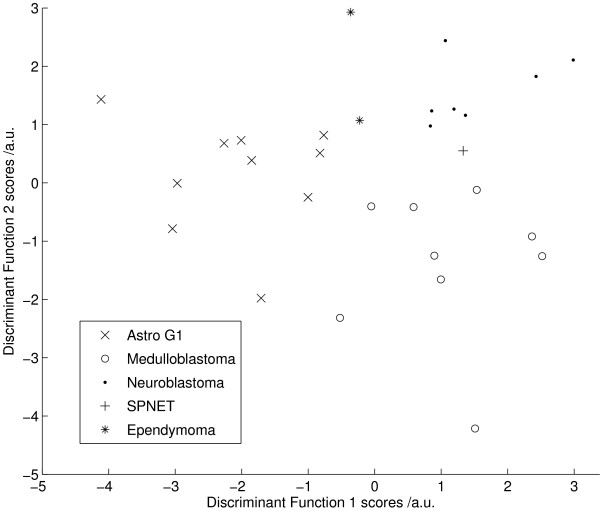
**LDA Astro vs MB**. Linear discriminant analysis of Astrocytoma G1 vs Medulloblastoma vs Neuroblastoma performed on the first 6 principal components of the fitted metabolite quantities. (classifier accuracy: 80%).

**Figure 6:**
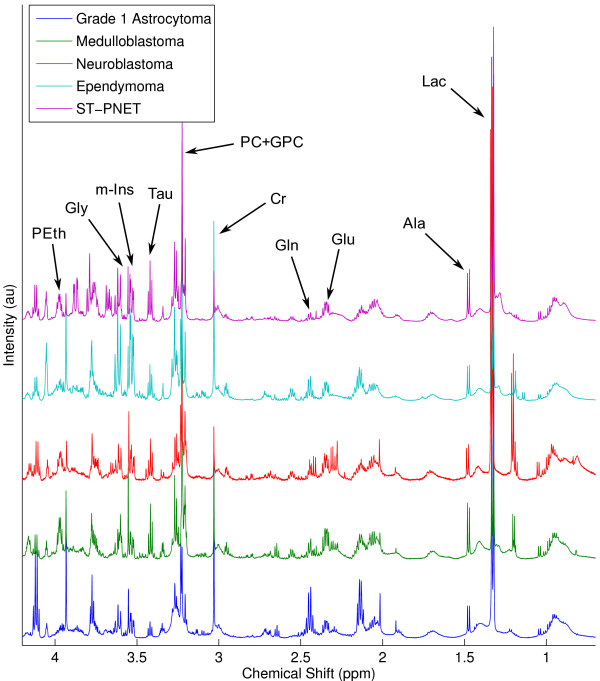
**HR-MAS spectra**. High resolution magic angle spinning 1H NMR spectra for the five tumour groups studied. Spectra were baseline corrected [36] and then scaled to the spectral area between 0.5 ppm and 4.5 ppm. Tau – Taurine, PEth – phosphorylethanolamine, PC – phosphorylcholine, m-Ins – myo-inositol, Lac – lactate, Gly – glycine, GPC – glycerophosphorylcholine, Gln – glutamine, Glu – glutamate, Cr – creatine.

Mean metabolite values for the three main tumour groups, medulloblastoma, neuroblastoma and pilocytic astrocytoma are given in Table 2. LDA was performed on the first 6 principal components from these tumour groups. From the scores plot of Figure 5, each tumour group is separable with no overlap. The classifier accuracy was 80% with an error rate of 32%. The first two discriminant functions were used to calculate scores for the ependymoma and supratentorial-PNET samples which were plotted on Figure 4. The ependymomas lie between the PNET and glial tumours whilst the supratentorial-PNET is between the neuroblastomas and the medulloblastomas. Typical spectra for the five tumour groups are shown in Figure 6.

## Discussion

In this study, metabolite profiles were measured using 1H HR-MAS on a series of ex vivo tissue samples from childhood brain and nervous system tumours. Metabolite profiles characteristic of PNETs and glial tumours were found and these provided a high accuracy of discrimination between the tumour groups. Notable differences were also detected between the two major contributors to the PNET group, medulloblastoma and neuroblastoma with a similarly high accuracy of discrimination.

An important finding in neuroblastomas is that metabolite concentrations generally vary less within a tumour than between tumours. Tumours can be highly heterogeneous at both the microscopic and the macroscopic level and it is important that a small area of tumour can be sampled without compromising the result. Furthermore, the small variability within the tumour is encouraging for the interpretation of in vivo MRS where large volumes (> 1 cm^3^) of tumour must be sampled for technical reasons. This finding is consistent with a report which compared in vivo MRS and 1H HR-MAS across a range of childhood brain tumours [23].

PCA is useful method for initial investigation of metabolite profiles since it reveals the characteristics which dominate the variability within the data. The separation between the glial and PNET tumours in the PCA (Figure 1) demonstrates that tissue morphology is a major determinant of metabolite profiles and that tumours with related histology have similar metabolite profiles. LDA finds the maximum separation between the groups and is useful for determining the relative contributions of individual metabolites in discriminating between the tumours. The LDA coefficients (Figure 2b) show that PNETs can be disciminated from glial tumours by higher taurine, phosphoethanolamine, phosphorylcholine and choline together with lower creatine and glutamine. This agrees with the findings of the 2 tailed t-test (Table 1) and confirms that these tumour metabolites define distinct metabolite profiles for these tumour groups. The PCA scores plot of Figure 3 shows that medulloblastomas have distinct metabolite profiles from neuroblastomas. These tumour groups can be difficult to distinguish on tissue morphology alone showing that tumour biology is also important in determining 1H HR-MAS metabolite profiles. Several studies have shown that medulloblastomas and neuroblastomas possess key molecular genetic markers and it has also been established that PNETs in the brain may be sub-categorized according to their gene expression profiles [4]. Evidence that 1H HR-MAS can detect subtle differences in metabolite profiles associated with tumour biology has previously been established in neuroblastoma cell lines [28]. It will now be important to use both 1H HR-MAS and molecular genetics to fully characterise a larger number of tumour samples and map the metabolite profiles to the molecular pathways involved. An understanding of the key pathways and their regulatory mechanisms is an important initial step in the discovery of novel tumour specific therapeutic targets.

The LDA performed on the three main tumour types, grade 1 astrocytomas, medulloblastomas and neuroblastoms (Figure 5) showed that ependymomas have features between those of pilocytic astrocytomas and PNETs whilst the supratentorial-PNET lay between the neuroblastomas and medulloblastomas. The analysis demonstrates that tumours with related histopathological features have similar metabolite profiles. This is to be expected for supratentorial-PNETs since they bear a close resemblance to other PNETs in both their morphology and behaviour although supratentorial-PNETs generally have a worse prognosis overall. However, ependymomas are very much more distinct from grade 1 astrocytomas and are clinically more aggressive. The groupings seen by the metabolite profiles therefore reflect the known biological and clinical behaviour of the tumours. This finding supports the grouping of the cases into glial tumours and PNETs for the purposes of building LDA classifiers. The combining of tumour types to form larger groups is an important strategy since paediatric tumours are rare and it is difficult to obtain sufficient numbers to build reliable classifiers for individual tumour types.

Rapid intra-operative diagnosis is an important clinical investigation and is currently carried out using frozen sections or smear preparations. 1H HR-MAS requires minimal sample preparation and the data can be acquired in less than 30 mins. When combined with automated software for spectral analysis and classification it has the potential to provide a preliminary diagnosis during surgery. Good quality data can be acquired from pieces of tissue smaller than 5 mg making it possible to acquire data on samples extracted via minimally invasive stereotactic biopsy although data acquisition times may be longer. Estimated diagnostic accuracies of 90% for the PNET versus glial tumour classifier and 94% for the neuroblastoma versus medulloblastoma classifier are encouraging. The lower accuracy achieved by the three tumour classifier (Figure 5) implies that the optimal strategy may be to use clinical and radiological information to reduce the number of diagnoses under consideration prior to the 1H HR-MAS analysis. Discriminating PNETs from glial tumours is clinically important for tumours in several locations, in particular the cerebellum and cerebral hemispheres. A prospective comparison with rapid histopathology techniques such as frozen sections would be an interesting extension of this work.

Biochemical changes have been noted previously in childhood brain and nervous system tumours but there is limited understanding of their significance. The high taurine in medulloblastomas has been reported previously in-vivo [29,30] and in small studies using 1H HR-MAS [22,27]. The current study shows that taurine is also high in neuroblastomas. Taurine is known to play an important role in neurodevelopment [31] and may be a marker of neural tumours, however, its role in tumourigenesis is unclear. Choline metabolism has been related to tumour growth in numerous studies reviewed in [32], and high phosphocholine/glycerophosphocholine ratio is seen in rapidly growing aggressive tumours [7]. The high phosphocholine and phosphocholine/glycerophosphocholine ratio seen in medulloblastomas is therefore consistent with their rapid growth and high grade and confirms data obtained on these tumours in vivo and ex vivo [27]. This pattern of metabolite values is not seen in neuroblastomas, however, 5 of the 7 tumours were MYCN non-amplified and it has been established in cell lines that phosphocholine/glycerophosphocholine is high in MYCN amplified but not MYCN non-amplified tumours [28]. Phosphocholine/glycerophosphocholine is low in pilocytic astrocytomas as expected from their clinical and biological properties. High phosphoethanolamine levels in medulloblastomas confirm an observation made in a prior 1H HR-MAS study [22] and may reflect the close relationship of this metabolite to the pathways for choline metabolism. Changes in glutamine levels may be mediated through its precursor glutamate. Glutamate is a key metabolite in the citric acid cycle and also an important neurotransmitter. In general, the biochemical changes found in these tumours are poorly understood and further studies elucidating the molecular pathways and their regulation are required to improve our understanding of these tumours.

## Conclusion

1H HR-MAS combined with automated spectral analysis is a powerful method for determining metabolite profiles of small tumour tissue samples. Specific differences exist between the 1H HR-MAS profiles of childhood glial and PNET tumours indicating that tumour morphology is an important determinant of metabolite profiles. Significant differences also exist between the metabolite profiles of medulloblastoma and neuroblastoma indicating that 1H HR-MAS can detect subtle differences between tumours which have closely related histopathology. Further elucidation of the key molecular pathways regulating these processes will improve our understanding of these tumours and may identify targets for new drugs tailored to specific tumours.

## Methods

### Patients and samples

Forty tissue samples were collected from 29 patients at Birmingham Childrens Hospital. Each sample was biopsied prior to the patient receiving treatment and a histopathological diagnosis being made. Ethical committee approval for the study and written informed consent from the family were obtained. The PNETs comprised 9 medulloblastomas, 7 neuroblastomas and 1 supratentorial PNET. Of the neuroblastomas: 4 had stage 1 disease and 3 had stage 4 disease; 2 tumours were MYCN amplified; 2 of the primary tumours were in the thorax whilst the rest were below the diaphragm; the patients ranged from 3 months to 38 months at diagnosis. The medulloblastomas were from 9 children, 1 had desmoplastic medulloblastoma and the rest had classic histology, 4 patients had localized tumours whilst 5 had metastatic disease (Chang stages 1 to 3). The glial tumours consisted of 10 pilocytic astrocytomas (grade 1) and 2 ependymomas. The pilocytic astrocytomas were in the cerebellum in 5 cases and in the supratentorial region in 5 cases. Both ependymomas arose from the cerebellum, one was a grade 2 tumour and the other a grade 3 tumour. The neuroblastoma cases had up to 3 samples available. The other tumour types had one sample available.

### 1H HR-MAS

Biopsy tissue was snap frozen in liquid nitrogen shortly after resection and stored at -80°C. Just prior to HR-MAS, tissue was thawed at room temperature and cut to approx 15 mg where appropriate. The mean sample mass was 10.2 mg with a standard deviation of 6.2 mg. The tissue was then placed into a 40 *μ*L wide mouth zirconia rotor and weighed. 4 *μ*L of 3-(trimethylsilyl)proponic-2,2,3,3-d4 acid sodium salt (TSP) was dissolved in D_2_O at a concentration of 10 mM and was added to the rotor. The remaining volume of the rotor was filled with D_2_O. 1H HR-MAS was performed on a Varian 600 MHz vertical bore spectrometer using a 4 mm gHX nanoprobe (Varian NMR Inc, Palo Alto, CA, USA) with a 3 channel INOVA console running VNMRj software. The probe temperature was set to 0.1°C to minimize sample degradation, and the sample was spun at 2500 Hz. This equated to a sample temperature of 6.7°C determined by methanol calibration. A standard pulse and acquire sequence was used which consisted of a single 90°C pulse preceded by one second of water presaturation. This was followed by the acquisition of 16 K complex points at a sampling frequency of 7200 Hz. 512 scans were acquired with a repetition time of 3.3 seconds giving a total acquisition time of 28 mins. A 30-ms CPMG pulse sequence was also used to aid metabolite assignment. This consisted of an xy 16 hard pulse train with a 1-s water presaturation pulse; the rotor speed was 2500 Hz; 512 scans were acquired with a repetition time of 3.3 seconds giving a total acquisition time of 28 mins. The phases of the CPMG refocussing pulses were arrayed (x, y, x, y, y, x, y, x,-x,-y,-x,-y,-y,-x,-y,-x) as described in [33]. Tuning and matching, 90° pulse width and the pre-saturation pulse frequency were optimised for each sample.

### Fitting and Multivariate Analysis

Raw data was Fourier transformed to 16 K points, phased and referenced to the creatine peak at 3.03 ppm using in house software. The phased data was then transformed back to the time-domain and the TARQUIN algorithm was used to fit the metabolite components of the signal [24]. This algorithm was chosen as it has been shown to be robust to the shifting of metabolite peaks caused by pH variation, which is of particular importance in the analysis of HR-MAS data.

The TARQUIN algorithm measures the metabolite quantities by fitting a series of simulated individual metabolite signals to the experimentally acquired data. The individual metabolite signals were simulated from chemical shift and j-coupling values published by Govindaraju et al [34]. Since the metabolite chemical shift values can have a minor dependence on temperature they were modified to match our experimental conditions to ensure the best starting point for the algorithm. Metabolite quantities were divided by the fitted spectral area between 0.5 and 4.5 ppm. Since multiple samples were available for some of the neuroblastoma tumours, an analysis of the intra-tumour versus inter-tumour metabolite variability was performed for these tumours using a z-test. The metabolite variability was defined as the ratio standard deviation/mean. Only tumours for which 3 samples were available were included in this analysis. All subsequent analysis used mean metabolite values for each tumour. A t-test was performed on all metabolite quantities to determine any significant differences between PNETs and glial tumours and between neuroblastoma and medulloblastoma profiles. Multivariate analysis was then performed following an approach developed for small MRS datasets and previously used to classify in-vivo MRS data [27]. Firstly, principal component analysis (PCA) was performed on the standardized metabolite quantities to provide an unsupervised analysis of the data and to reduce the effective number of variables used in the subsequent supervised analysis. Linear discriminant analysis (LDA) was then carried out on the principal component scores using a leave-one-out (LOO) process to estimate the classification accuracy. The number of principal components included in the LDA was selected to be the smallest of: a) the number of components required to explain 90% of the variance present in the data, b) the number of samples in the smallest group minus one, c) the minimum number of components required to achieve optimum classification accuracy. Since the classification error estimated in this way is susceptible to bias from over-fitting, cross validation using the 632+ bootstrapping method, as described by Davies et al [27], was used to estimate the error eB632+.

This method was used to classify samples in two groups as either glial type tumours or PNETs. A second classifier was then developed to separate the PNET samples as medulloblastomas and neuroblastomas. To compare the profiles of each individual tumour type, a three group classifier was developed to distinguish astrocytoma grade 1 tumours, medulloblastomas and neuroblastomas. The supratentorial-PNET and ependymoma tumours were excluded for the calculation of the disciminant coefficients in the three group classifier to prevent over – fitting for groups with low sample numbers. Once disciminant coefficients had been calculated, they were then used to calculate the scores for the full data set including supratentorial-PNET and ependymoma samples. The MATLAB [35] Statistics Toolbox implementation of PCA and LDA was used.

## Competing interests

The authors declare that they have no competing interests.

## Authors' contributions

MW – Drafted the paper, collected, processed and analysed the HR-MAS data. ND – Performed the multivariate analysis and classification of the data and assisted in the interpretation of its results. CM, MB – Were involved in the collection and verification of tissue samples and clinical details. RG, AP – Conceived and designed the study and assisted in the interpretation of results. All authors have read and approved the contents.
